# Sexually dimorphic effect of aging on skeletal muscle protein synthesis

**DOI:** 10.1186/2042-6410-3-11

**Published:** 2012-05-23

**Authors:** Gordon I Smith, Dominic N Reeds, Angela M Hall, Kari T Chambers, Brian N Finck, Bettina Mittendorfer

**Affiliations:** 1 Division of Geriatrics and Nutritional Science, Washington University School of Medicine, 660 South Euclid Avenue; Campus, Box 8031, Saint Louis, MO 63110, USA

**Keywords:** Muscle protein turnover, Aging, Sarcopenia, Amino acid

## Abstract

**Background:**

Although there appear to be no differences in muscle protein turnover in young and middle aged men and women, we have reported significant differences in the rate of muscle protein synthesis between older adult men and women. This suggests that aging may affect muscle protein turnover differently in men and women.

**Methods:**

We measured the skeletal muscle protein fractional synthesis rate (FSR) by using stable isotope-labeled tracer methods during basal postabsorptive conditions and during a hyperaminoacidemic-hyperinsulinemic-euglycemic clamp in eight young men (25–45 y), ten young women (25–45 y), ten old men (65–85 y) and ten old women (65–85 y).

**Results:**

The basal muscle protein FSR was not different in young and old men (0.040 ± 0.004 and 0.043 ± 0.005%·h^-1^, respectively) and combined insulin, glucose and amino acid infusion significantly increased the muscle protein FSR both in young (to 0.063 ± 0.006%·h^-1^) and old (to 0.051 ± 0.008%·h^-1^) men but the increase (0.023 ± 0.004 vs. 0.009 ± 0.004%·h^-1^, respectively) was ~60% less in the old men (P = 0.03). In contrast, the basal muscle protein FSR was ~30% greater in old than young women (0.060 ± 0.003 vs. 0.046 ± 0.004%·h^-1^, respectively; P < 0.05) and combined insulin, glucose and amino acid infusion significantly increased the muscle protein FSR in young (P < 0.01) but not in old women (P = 0.10) so that the FSR was not different between young and old women during the clamp (0.074 ± 0.006%·h^-1^ vs. 0.072 ± 0.006%·h^-1^, respectively).

**Conclusions:**

There is sexual dimorphism in the age-related changes in muscle protein synthesis and thus the metabolic processes responsible for the age-related decline in muscle mass.

## Background

Understanding how aging affects muscle protein metabolism is important in order to devise adequate countermeasures for the age-related loss of muscle mass. It is well known that there is sexual dimorphism with regards to body composition. Healthy adult women have less lean body and muscle mass and more fat than men [1-3] and the age-related decrease in muscle mass is slower in women than in men [1,4-6]. Insight into the mechanism(s) responsible for these differences in phenotype is limited, however. Several studies indicate that there is no difference in the basal rate of muscle protein synthesis [7-11] or muscle protein breakdown [8] or the anabolic responses to nutritional stimuli [11] and resistance exercise [7] in young and middle-aged adult men and women. On the other hand, we have recently found that the basal rate of muscle protein synthesis is greater in obese, old women than in obese, old men [12]. In addition, we found that obese, old women, but not obese, old men, failed to significantly increase the rate of muscle protein synthesis in response to mixed meal ingestion [12]. This suggests that differences in muscle protein turnover between men and women might be most apparent when muscle mass is changing (i.e., during aging vs. earlier adulthood when muscle mass is steady) and that aging affects muscle protein turnover differently in men and women. To our knowledge, only one study so far has evaluated the effect of both sex and aging on basal muscle protein turnover [13]. However, this study was limited to basal, postabsorptive conditions only and included only old men with hypogonadism and old women who had very low serum androgen concentrations, which may have confounded the results. Hypoandrogenemia is associated with a reduced lean body mass [14] and treatment with testosterone increases the muscle protein synthesis rate [15-20].

The primary purpose of our study therefore was to evaluate the effect of aging on the basal rate of muscle protein synthesis and the anabolic response to combined hyperaminoacidemia and hyperinsulinemia in men and women. We hypothesized that: i) the anabolic response to increased amino acid and insulin availability would be reduced in old compared with young subjects (both men and women), ii) the age-related decline in the anabolic response would be greater in women than in men, and iii) the basal rate of muscle protein synthesis would be greater in old compared with young women. We also measured the concentrations of myostatin and follistatin in plasma and the expression of the genes encoding myostatin, myoD and follistatin in muscle to gain information of potential differences in cellular factors that regulate protein synthesis in men and women and young and old subjects. Myostatin is a muscle growth inhibitor which is produced primarily in skeletal muscle cells, circulates in the blood and acts on muscle tissue by blocking genes induced during differentiation (e.g., myoD and myogenin, which are myogenic growth factors [21]) and by inhibiting the anabolic signaling cascade and muscle protein synthesis [22-26]. Follistatin is ubiquitously expressed, circulates in the blood and binds to and thereby inhibits myostatin [27,28]. We therefore hypothesized that: i) muscle myostatin gene expression and myostatin concentration in plasma would be greater in old than young subjects and greater in old men than old women whereas ii) muscle myoD and follistatin mRNA expression and plasma follistatin concentration would be greater in young than old subjects and greater in old women than old men.

## Methods

### Subjects

Thirty-eight non-obese subjects (8 men and 10 women who were between 25 and 45 y old and 10 men and 10 women who were between 65 and 85 y old) participated in this study. Data from 8 young men and 8 young women have previously been reported [11]. None of the subjects engaged in regular physical activities (i.e., they exercised ≤1.5 h·wk^-1^) or took medications (including hormonal contraceptives or hormone replacement therapy), and none reported excessive alcohol intake or consumed tobacco products. All subjects were considered to be in good health after completing a comprehensive medical evaluation, which included a history and physical examination, standard blood tests, and an oral glucose (75 g) tolerance test (Table 1). Written informed consent was obtained from all subjects before their participation in the study, which was approved by the Human Research Protection Office at Washington University School of Medicine in St. Louis, MO.

**Table 1:** Subjects’ anthropometric and basic metabolic characteristics at the time of screening

	**MEN**		**WOMEN**		**ANOVA**		
	***Young***	***Old***	***Young***	***Old***	***Sex***	***Age***	***Interaction***
Age (years)	40 ± 2	69 ± 1	37 ± 2	73 ± 2	0.84	<0.001	0.10
Body mass (kg)	81 ± 4	81 ± 3	69 ± 2	61 ± 4	<0.001	0.22	0.25
Body mass index (kg/m^2^)	26.5 ± 1.0	25.9 ± 0.8	25.0 ± 0.8	24.0 ± 1.3	0.09	0.44	0.84
Fat mass (kg)	18 ± 2	21 ± 2	22 ± 1	23 ± 3	0.16	0.34	0.61
Fat mass (% body mass)	21 ± 2	25 ± 2	32 ± 1	36 ± 2	<0.001	0.055	0.97
Fat free mass (kg)	63 ± 2	60 ± 2	47 ± 2	38 ± 1	<0.001	0.002	0.17
Fat free mass (% body mass)	79 ± 2	75 ± 2	68 ± 1	64 ± 2	<0.001	0.055	0.97
Appendicular muscle mass (kg)	27.5 ± 1.1	24.9 ± 0.7	18.0 ± 0.9	14.1 ± 0.5	<0.001	<0.001	0.46
Appendicular muscle mass index (kg/m^2^)	9.0 ± 0.2	8.0 ± 0.2	6.5 ± 0.2	5.5 ± 0.2	<0.001	<0.001	0.99
Fasting plasma glucose (mg/dl)	93 ± 1	95 ± 3	87 ± 1	90 ± 1	0.005	0.18	0.54
2 h post OGTT plasma glucose (mg/dl)	94 ± 7	111 ± 8	93 ± 4	106 ± 7	0.66	0.03	0.77
HOMA-IR	1.46 ± 0.32	1.59 ± 0.27	1.08 ± 0.22	1.16 ± 0.23	0.13	0.69	0.91
Systolic blood pressure (mm Hg)	109 ± 3	119 ± 5	105 ± 3	126 ± 6	0.69	<0.001	0.21
Diastolic blood pressure (mm Hg)	69 ± 2	74 ± 3	65 ± 3	68 ± 4	0.14	0.21	0.70
Plasma triglycerides (mg/dl)	88 ± 18	102 ± 12	66 ± 8	72 ± 13	0.04	0.43	0.75
Total plasma cholesterol (mg/dl)	170 ± 10	193 ± 7	172 ± 8	197 ± 8	0.74	0.01	0.89
LDL-cholesterol (mg/dl)	106 ± 9	125 ± 6	97 ± 7	109 ± 8	0.09	0.03	0.61
HDL-cholesterol (mg/dl)	47 ± 4	49 ± 3	62 ± 4	73 ± 4	<0.001	0.09	0.25
Testosterone (ng/ml)	4.8 ± 0.5	5.7 ± 0.5	0.9 ± 0.1	0.8 ± 0.1	<0.001	0.26	0.18
Estradiol (pg/ml)	25 ± 2	18 ± 2	62 ± 9^a^	8 ± 3^b^	0.01	<0.001	<0.001
Progesterone (ng/ml)	0.24 ± 0.10	0.28 ± 0.08	5.08 ± 1.53^a^	0.23 ± 0.07	<0.01	<0.01	<0.01

Values are means ± SEM. HOMA-IR: Homeostasis model assessment of insulin resistance. OGTT: oral glucose tolerance test.

^a^ Value significantly different from values in men and old women (*P* < 0.01). ^b^ Value significantly different from values in men (*P* < 0.05).

### Experimental protocol

Approximately two weeks before the protein metabolism study, subjects' total body mass, fat mass, fat-free mass (FFM) and appendicular muscle mass (Table 1) were measured by using dual-energy X-ray absorptiometry (Delphi-W densitometer, Hologic, Waltham, MA) [29]. The appendicular muscle mass index, a measure of muscle mass adjusted for individual differences in height was calculated by dividing total appendicular muscle mass (kg) by height squared (m^2^) [30]. Subjects were instructed to adhere to their usual diet and to refrain from vigorous physical activities for three days before the protein metabolism study. We did not control for menstrual cycle phase in our young women because Miller et al. [31] demonstrated that the rate of muscle protein synthesis is not different during the follicular and luteal phases of the menstrual cycle and we [11] have found that there is no relationship between plasma estradiol or progesterone concentrations and the muscle protein FSR in young women. The evening before the study, subjects were admitted to the Clinical Research Unit at Washington University School of Medicine. At 2000 h, they consumed a standard meal providing 50.2 kJ per kg body weight (15% as protein, 55% as carbohydrates and 30% as fat). Subjects then rested in bed and fasted (except for water) until completion of the study the next day. At ~0600 h on the following morning, a cannula was inserted into a vein in the forearm or the antecubital fossa of one arm for the infusion of stable isotope labeled tracers, insulin, glucose, and amino acids; another cannula was inserted into a vein of the contralateral hand (which was warmed to 55°C) to obtain arterialized blood samples. At ~0800 h, primed, constant infusions of [ring-^2^ H_5_phenylalanine (priming dose: 2.8 μmol·kg FFM^-1^, infusion rate: 0.08 μmol·kg FFM^-1^·min^-1^) and [6,6-^2^ H_2_glucose (priming dose: 18 μmol·kg body wt^-1^, infusion rate: 0.22 μmol·kg body wt^-1^·min^-1^), both purchased from Cambridge Isotope Laboratories Inc. (Andover, MA), were started and maintained for seven hours. Four hours after the start of the tracer infusions, a hyperinsulinemic-hyperaminoacidemic-euglycemic clamp was started and maintained for three hours. Human insulin (Novolin R, Novo Nordisk, Princeton, NJ) was infused at a rate of 20 mU·m^-2^ body surface area (BSA)·min^-1^ (initiated with priming doses of 80 mU·m^-2^ BSA·min^-1^ for the initial 5 minutes and 40 mU·m^-2^ BSA·min^-1^ for an additional 5 minutes). Plasma amino acid availability was increased by providing an intravenous amino acid mixture (Travasol 10%, Baxter, Deerfield, IL) infused at a rate of 105 mg amino acids·kg FFM^-1^·h^-1^ (priming dose: 35 mg amino acids·kg FFM^-1^). During the insulin infusion, euglycemia at a blood glucose concentration of ~5.5 mM was maintained by variable rate infusion of 20% dextrose solution (Baxter, Deerfield, IL) which was enriched (2.5%) with [6,6-^2^ H_2_glucose. To adjust for the increased plasma amino acid availability and reduced hepatic glucose production during the clamp procedure, the [ring-^2^ H_5_phenylalanine and [6,6-^2^ H_2_glucose infusion rates were increased to 0.12 μmol·kg FFM^-1^·min^-1^(phenylalanine) and decreased to 0.11 μmol·kg body wt^-1^ min^-1^ (glucose), respectively.

Blood samples (~3 ml each) were obtained before beginning the tracer infusion and at 60, 90, 180, 210, 220, 230, 240, 270, 300, 330, 360, 390, 400, 410, and 420 min to determine phenylalanine and glucose tracer-to-tracee ratios (TTR) in plasma and plasma concentrations of insulin, glucose, myostatin, follistatin, phenylalanine, and leucine (thought to be a major regulator of muscle protein synthesis [32]). Additional blood samples (~1 ml each) were obtained every 10 minutes during the clamp procedure to monitor plasma glucose concentration. Muscle tissue (~50-100 mg) was obtained under local anesthesia (lidocaine, 2%) from the quadriceps femoris by using a Tilley-Henkel forceps [33] at 1 h, 4 h and 7 h after starting the tracer infusion to determine the muscle protein fractional synthesis rate (FSR) during basal conditions (1 h – 4 h) and during the hyperinsulinemic-hyperaminoacidemic-euglycemic clamp (4 h – 7 h) and the mRNA expressions (initial biopsy at 1 h only) of myostatin, myoD, and follistatin.

### Sample processing and analyses

To determine plasma glucose concentration, blood was collected in pre-chilled tubes containing heparin, plasma was separated immediately by centrifugation and glucose concentration was measured immediately. All other blood samples were collected in pre-chilled tubes containing EDTA, plasma was separated by centrifugation within 30 min of collection and then stored at −80°C until final analyses. Muscle samples were rinsed in ice-cold saline immediately after collection, cleared of visible fat and connective tissue, frozen in liquid nitrogen and stored at −80°C until final analyses were performed.

Plasma glucose concentration was measured on an automated glucose analyzer (Yellow Spring Instruments, Yellow Springs, OH). Plasma insulin concentration was determined by radioimmunoassay (Linco Research, St. Louis, MO). Commercially available ELISA kits were used to determine the concentrations of testosterone, estradiol, progesterone (all IBL America, Minneapolis, MN), myostatin (ALPCO Diagnostics, Salem, NH) and follistatin (R&D Systems, Minneapolis, MN) in plasma.

To determine the labeling of plasma glucose, plasma proteins were precipitated with ice-cold acetone, and lipids were extracted with hexane. The aqueous phase, containing glucose, was dried by speed-vac centrifugation (Savant Instruments, Farmingdale, NY), glucose was derivatized with heptafluorobutyric acid and the TTR was determined by using gas-chromatography/mass-spectrometry (GC-MS, Hewlett-Packard MSD 5973 system with capillary column) as previously described [34].

To determine plasma concentrations of leucine and phenylalanine and the labeling of plasma phenylalanine, known amounts of nor-leucine and [1-^13^ C]phenylalanine were added to an aliquot of each plasma sample, plasma proteins were precipitated, and the supernatant, containing free amino acids, was collected to prepare the *t*-butyldimethylsilyl (*t*-BDMS) derivative of leucine and phenylalanine to determine their TTRs by GC-MS (MSD 5973 System, Hewlett-Packard) [35,36]. To determine phenylalanine labeling in muscle proteins and in tissue fluid, samples (~20 mg) were homogenized in 1 ml trichloroacetic acid solution (3% w/v), proteins were precipitated by centrifugation, and the supernatant, containing free amino acids, was collected. The pellet containing muscle proteins was washed and then hydrolyzed in 6 N HCl at 110°C for 24 h. Amino acids in the protein hydrolysate and supernatant samples were purified on cation-exchange columns (Dowex 50 W-X8-200, Bio-Rad Laboratories, Richmond, CA), and the t-BDMS derivative of phenylalanine prepared to determine its TTR by GC-MS (MSD 5973 System, Hewlett-Packard) analysis [35,36]. The extent of phenylalanine labeling in plasma (from arterialized blood samples), muscle tissue fluid, and muscle protein were calculated based on the simultaneously measured TTR of standards of known isotope labeling.

Muscle myostatin, myoD and follistatin gene expression was evaluated by using RT-PCR. RNA was isolated in RNA-Bee reagent (Tel-Test, Inc, Friendswood, TX), quantified spectrophotometrically (NanoDrop 1000, Thermo Scientific, Waltham, MA) and reverse transcribed (Taqman Reverse Transcription Kit, Applied Biosystems, Foster City, CA) by using the SYBR Green Master Mix (Applied Biosystems, Carlsbad, CA) on an ABI 7500 real-time PCR system (Applied Biosystems, Carlsbad, CA) using the following primer sequences (all 5' to 3'). Myostatin forward: ACC TGT TTA TGC TGA TTG TTG CT, reverse: GAG CTG TTT CCA GAC GAA GTT TA. MyoD forward: CGC CAT CCG CTA TAT CGA GG, reverse: CTG TAG TCC ATC ATG CCG TCG. Follistatin forward: GTA ATC GGA TTT GCC CAG AGC, reverse: GCA GGC AGG TAG CCT TTC T. Results were normalized to the 36B4 housekeeping gene.

### Calculations

The muscle protein FSR was calculated from the rate of [ring-^2^ H_5_phenylalanine incorporation into muscle protein, using a standard precursor-product model as follows: FSR = ΔE_p_/E_ic_ × 1/*t* × 100; where ΔE_p_ is the change between two consecutive biopsies in extent of labeling (TTR) of protein-bound phenylalanine. E_ic_ is the mean labeling over time of the precursor for protein synthesis and *t* is the time between biopsies. The free phenylalanine labeling in muscle tissue fluid was chosen to represent the immediate precursor for muscle protein synthesis (i.e., aminoacyl-*t*-RNA) [37].

Glucose rates of appearance (Ra) in plasma during basal conditions and during the clamp procedure were calculated by dividing the glucose tracer infusion rate by the average plasma (from arterialized blood samples) glucose TTR during the last 30 min of the basal period and the last 30 min of the clamp, respectively. Glucose Ra during basal conditions represents endogenous glucose Ra and thus an index of hepatic glucose production rate. During the clamp procedure, glucose Ra represents the sum of endogenous glucose Ra and the rate of infused glucose. Endogenous glucose Ra during the clamp was therefore calculated by subtracting the glucose infusion rate from glucose Ra; glucose rate of disappearance (Rd) was assumed to be equal to glucose Ra plus the tracer infusion rate. The homeostasis model assessment of insulin resistance (HOMA-IR) score was calculated by dividing the product of basal glucose and insulin concentrations (expressed in mM and mIU/l, respectively) by 22.5 [38].

### Statistical analysis

**Figure 1:**
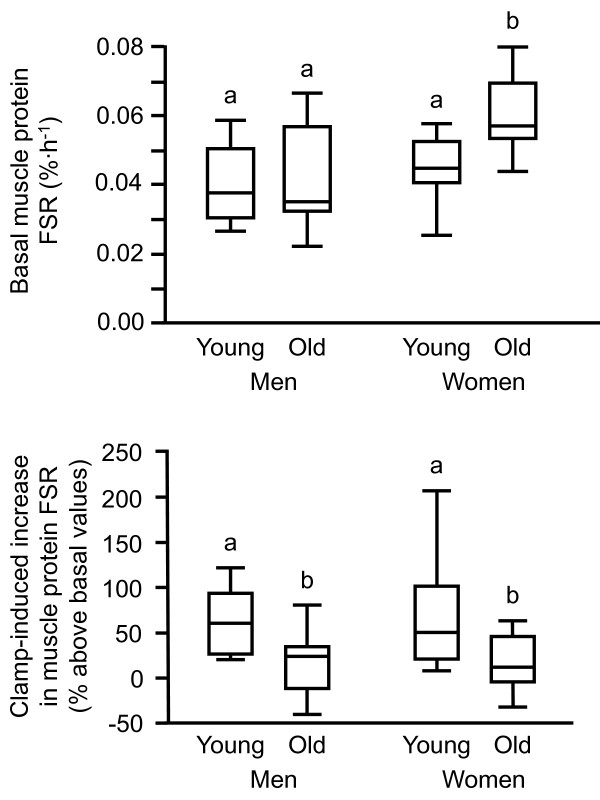
**Skeletal muscle protein fractional synthesis rate (FSR) during basal, post-absorptive conditions (top) and the increase in muscle protein FSR in response to the hyperinsulinemic-hyperaminoacidemic-euglycemic clamp procedure (bottom) in young and old men and young and old women.** Data are median (central horizontal line), inter-quartile range (box), and minimum and maximum values (vertical lines). Bars not sharing the same letter are significantly different from each other (P < 0.05).

All data sets were normally distributed. Two-way analysis of variance (ANOVA; with age and study condition, i.e., basal vs. clamp as factors) was used to compare the muscle protein FSR, and substrate and hormone concentrations in young and old men and in young and old women, respectively. In addition, 2-way ANOVA with age and sex as factors was used to compare the basal muscle protein FSR, the anabolic response to increased amino acid and insulin availability, plasma substrate, hormone and myogenic regulatory factor concentrations, and muscle gene expression amongst all four groups (young men, old men, young women, and old women). When significant interactions were found, Tukey’s post-hoc procedure was used to locate the differences. A *P*-value of ≤0.05 was considered statistically significant. Data are presented as means ± SEM unless otherwise noted (i.e., Figure 1). Statistical analyses were carried out by using the PASW statistical software package 18 (IBM, Armonk, NY).

## Results

### Plasma hormone, glucose and amino acid concentrations

Plasma testosterone concentration was significantly greater in men than women (P < 0.001) and was not affected by aging (Table 1). Plasma progesterone concentration was significantly lower in men and old women than in young women (P < 0.01). Plasma estradiol concentration was greatest in young women (P < 0.01 compared with all other groups) and lowest in old women (P < 0.01 vs. young and old men and young women) (Table 1).

Aging had no effect on plasma glucose, insulin, leucine and phenylalanine concentrations – neither during basal, postabsorptive conditions nor during combined insulin, glucose and amino acid infusion (Tables 2, 3 and 4). Plasma glucose, insulin and phenylalanine concentrations were not different between men and women but plasma leucine concentration was ~15% (P <0.01) greater in men than in women (Tables 2, 3 and 4).

**Table 2:** Plasma glucose, insulin, leucine and phenylalanine concentrations during basal, postabsorptive conditions and during the hyperinsulinemic-hyperaminoacidemic-euglycemic clamp procedure

	**Young**		**Old**	
	**Basal**	**Clamp**	**Basal**	**Clamp**
MEN				
Glucose (mM)	5.0 ± 0.1	5.4 ± 0.1^a^	5.1 ± 0.1	5.4 ± 0.1^a^
Insulin (mU·l^-1^)	6.5 ± 1.4	29.1 ± 2.0^a^	7.1 ± 1.2	31.3 ± 3.2^a^
Leucine (μM)	123 ± 7	166 ± 12^a^	118 ± 7	176 ± 10^a^
Phenylalanine (μM)	56 ± 7	98 ± 12^a^	64 ± 8	111 ± 12^a^
WOMEN				
Glucose (mM)	4.8 ± 0.1	5.4 ± 0.1^a^	4.9 ± 0.1	5.5 ± 0.1^a^
Insulin (mU·l^-1^)	5.2 ± 1.2	32.3 ± 5.9^a^	5.2 ± 1.0	32.3 ± 2.2^a^
Leucine (μM)	99 ± 7^b^	131 ± 12^a,b^	104 ± 5^b^	137 ± 9^a,b^
Phenylalanine (μM)	61 ± 3	119 ± 10^a^	61 ± 3	109 ± 4^a^

Values are mean ± SEM. ^a^ Value significantly different from corresponding value during basal conditions (*P* < 0.001). ^b^ Value significantly different from corresponding value in men (*P* < 0.01).

**Table 3:** Plasma phenylalanine concentrations and enrichments and muscle free and protein-bound phenylalanine enrichments during basal, postabsorptive conditions and during the hyperinsulinemic-hyperaminoacidemic clamp in young and old men

**Time (min)**	**Concentration**		**Enrichment**					
	**(μM)**		**Muscle protein bound TTR**		**Muscle free TTR**		**Plasma TTR**	
	***Young***	***Old***	***Young***	***Old***	***Young***	***Old***	***Young***	***Old***
Basal conditions								
60	59 ± 8	64 ± 9	0.000033 ± 0.000007	0.000101 ± 0.000026	0.0488 ± 0.0037	0.0545 ± 0.0044	0.0921 ± 0.0055	0.0929 ± 0.0066
90	48 ± 6	67 ± 8	---	---	---	---	0.1030 ± 0.0077	0.0993 ± 0.0090
120	58 ± 9	63 ± 8	---	---	---	---	0.1032 ± 0.0056	0.1119 ± 0.0093
150	53 ± 9	61 ± 9	---	---	---	---	0.1118 ± 0.0119	0.1130 ± 0.0067
180	56 ± 10	63 ± 9	---	---	---	---	0.1088 ± 0.0034	0.1129 ± 0.0056
210	60 ± 7	66 ± 8	---	---	---	---	0.1093 ± 0.0031	0.1155 ± 0.0078
240	59 ± 8	64 ± 9	0.000099 ± 0.000009	0.000186 ± 0.000029	0.0589 ± 0.0027	0.0686 ± 0.0048	0.1065 ± 0.0032	0.1194 ± 0.0079
**Δ (240 – 60 min)**			**0.000067 ± 0.000009**	**0.000085 ± 0.000013**				
**Mean**	**56 ± 7**	**64 ± 8**			**0.0539 ± 0.0028**	**0.0615 ± 0.0041**	**0.1050 ± 0.0051**	**0.1093 ± 0.0071**
Hyperinsulinemic-hyperaminoacidemic clamp								
270	93 ± 14	97 ± 10	---	---	---	---	0.0969 ± 0.0031	0.1108 ± 0.0045
300	88 ± 12	97 ± 10	---	---	---	---	0.0958 ± 0.0034	0.1028 ± 0.0042
330	116 ± 18	107 ± 14	---	---	---	---	0.0942 ± 0.0022	0.1005 ± 0.0050
360	85 ± 10	121 ± 14	---	---	---	---	0.0950 ± 0.0025	0.1000 ± 0.0044
390	98 ± 12	128 ± 16	---	---	---	---	0.0937 ± 0.0013	0.1021 ± 0.0055
420	108 ± 14	116 ± 17	0.000235 ± 0.000023	0.000310 ± 0.000038	0.0719 ± 0.0030	0.0806 ± 0.0033	0.0960 ± 0.0019	0.0952 ± 0.0055
**Δ (420 – 240 min)**			**0.000136 ± 0.000015**	**0.000124 ± 0.000020**				
**Mean**	**98 ± 12**	**111 ± 12**			**0.0654 ± 0.0027**	**0.0746 ± 0.0038**	**0.0953 ± 0.0021**	**0.1019 ± 0.0043**

Values are means ± SEM.

**Table 4:** Plasma phenylalanine concentrations and enrichments and muscle free and protein-bound phenylalanine enrichments during basal, postabsorptive conditions and during the hyperinsulinemic-hyperaminoacidemic clamp in young and old women

**Time (min)**	**Concentration**		**Enrichment**					
	**(μM)**		**Muscle protein bound TTR**		**Muscle free TTR**		**Plasma TTR**	
	***Young***	***Old***	***Young***	***Old***	***Young***	***Old***	***Young***	***Old***
Basal conditions								
60	63 ± 3	60 ± 3	0.000057 ± 0.000015	0.000062 ± 0.000017	0.0602 ± 0.0032	0.0622 ± 0.0052	0.0906 ± 0.0038	0.0862 ± 0.0027
90	64 ± 6	61 ± 2	---	---	---	---	0.0986 ± 0.0048	0.0893 ± 0.0039
120	56 ± 3	54 ± 2	---	---	---	---	0.1009 ± 0.0015	0.0952 ± 0.0040
150	56 ± 5	64 ± 3	---	---	---	---	0.0998 ± 0.0031	0.0967 ± 0.0043
180	59 ± 2	60 ± 4	---	---	---	---	0.1038 ± 0.0034	0.1013 ± 0.0038
210	63 ± 3	62 ± 4	---	---	---	---	0.1041 ± 0.0029	0.1020 ± 0.0043
240	66 ± 2	65 ± 4	0.000147 ± 0.000015	0.000176 ± 0.000020	0.0722 ± 0.0040	0.0673 ± 0.0045	0.1023 ± 0.0024	0.1026 ± 0.0039
**Δ (240 – 60 min)**			**0.000090 ± 0.000014**	**0.000114 ± 0.000012**				
**Mean**	**61 ± 3**	**61 ± 3**			**0.0662 ± 0.0035**	**0.0648 ± 0.0044**	**0.1000 ± 0.0027**	**0.0962 ± 0.0035**
Hyperinsulinemic-hyperaminoacidemic clamp								
270	111 ± 10	92 ± 4	---	---	---	---	0.1027 ± 0.0045	0.1013 ± 0.0086
300	108 ± 8	111 ± 7	---	---	---	---	0.0950 ± 0.0024	0.1026 ± 0.0065
330	122 ± 16	106 ± 7	---	---	---	---	0.0926 ± 0.0020	0.0894 ± 0.0069
360	124 ± 14	122 ± 9	---	---	---	---	0.0944 ± 0.0032	0.0922 ± 0.0035
390	128 ± 11	109 ± 5	---	---	---	---	0.0970 ± 0.0029	0.0838 ± 0.0063
420	120 ± 5	114 ± 3	0.000305 ± 0.000020	0.000348 ± 0.000027	0.0752 ± 0.0019	0.0782 ± 0.0033	0.0971 ± 0.0026	0.0929 ± 0.0038
**Δ (420 – 240 min)**			**0.000157 ± 0.000016**	**0.000171 ± 0.000017**				
**Mean**	**119 ± 10**	**109 ± 4**			**0.0737 ± 0.0028**	**0.0727 ± 0.0034**	**0.0965 ± 0.0024**	**0.0937 ± 0.0046**

Values are means ± SEM.

### Plasma myostatin and follistatin concentrations and muscle myoD, myostatin and follistatin gene expression

Plasma myostatin concentration was not different in men and women and was not affected by aging (Table 5). Plasma follistatin concentration was ~30% greater in old compared with young subjects but was not significantly different in men and women (Table 5). Muscle myoD, myostatin, and follistatin mRNA expressions were not affected by age or sex (Table 5).

**Table 5:** Plasma follistatin and myostatin concentrations and muscle myoD, myostatin, and follistatin gene expression in young and old men and women

	**MEN**		**WOMEN**		**ANOVA**		
	**Young**	**Old**	**Young**	**Old**	**Sex**	**Age**	**Interaction**
Plasma (ng/mL)							
Myostatin	3.12 ± 0.30	3.36 ± 0.24	3.29 ± 0.34	3.54 ± 0.42	0.61	0.47	0.99
Follistatin	1.58 ± 0.12	1.87 ± 0.12	1.22 ± 0.08	1.81 ± 0.12	0.07	0.005	0.19
Muscle mRNA (arbitrary units)							
Myostatin	1.00 ± 0.28	0.73 ± 0.17	0.82 ± 0.12	0.95 ± 0.17	0.92	0.70	0.28
Follistatin	1.00 ± 0.32	0.68 ± 0.19	1.40 ± 0.46	1.55 ± 0.59	0.15	0.85	0.58
MyoD	1.00 ± 0.39	1.21 ± 0.21	1.26 ± 0.38	1.12 ± 0.28	0.68	0.97	0.68

Values are means ± SEM. Muscle mRNA data were obtained from muscle biopsies collected during basal conditions.

### Muscle protein synthesis

**Figure 2:**
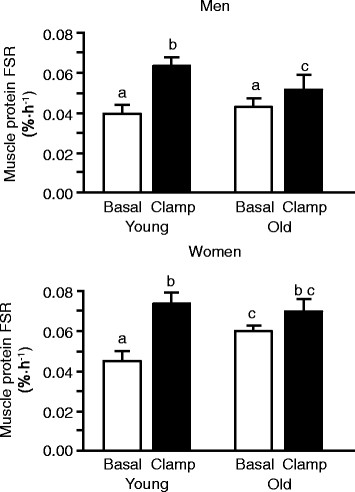
**Skeletal muscle protein fractional synthesis rate (FSR) during basal, post-absorptive conditions and during the hyperinsulinemic-hyperaminoacidemic-euglycemic clamp procedure in young and old men (top) and young and old women (bottom).** Data are means ± SEM. For both men and women, ANOVA revealed a significant age x clamp interaction (P < 0.05). Within each group (i.e., men or women), bars not sharing the same letter are significantly different from each other (P < 0.05).

The basal, postabsorptive muscle protein FSR was not different in young and old men and young women (0.040 ± 0.004, 0.043 ± 0.005, and 0.046 ± 0.004%·h^-1^, respectively) but was ~30% greater in old women (0.060 ± 0.003%·h^-1^) than in young and old men and young women (Figure 1, top panel and Figure 2; P < 0.05). Combined insulin, glucose and amino acid infusion significantly increased the muscle protein FSR both in young and old men but the increase was ~60% less in the old men (P < 0.05); consequently, the muscle protein FSR during the clamp was significantly greater (P < 0.01) in young than in old men (Figure 2, top panel). Combined insulin, glucose and amino acid infusion also significantly increased the muscle protein FSR in young (P < 0.01) but not in old women; consequently, the muscle protein FSR during the clamp was not different between young and old women (Figure 2, bottom panel). Overall, the anabolic response (increase in muscle protein FSR above basal values) was greater in young than old subjects but was not different in young men and young women and not different in old men and old women (Figure 1, bottom panel).

### Glucose kinetics

Basal glucose Ra was not different in young and old subjects but was significantly lower in men compared with women (9.4 ± 0.3 and 9.6 ± 0.3 μmol·kg body wt^-1^·min^-1^ in young and old men; 9.9 ± 0.4 and 11.3 ± 0.6 μmol·kg body wt^-1^·min^-1^ in young and old women; main effect of sex; P < 0.05). During the clamp, glucose Ra decreased (P < 0.01) to the same extent in all groups (i.e., by 71 ± 3, 70 ± 4, 73 ± 5, and 68 ± 6% in young and old men, and young and old women, respectively). The insulin mediated increase in glucose Rd during the clamp (144 ± 17, 133 ± 23, 168 ± 18 and 145 ± 22% in young and old men and young and old women, respectively) was also not affected by age (P = 0.44) or sex (P = 0.40).

## Discussion

Nutritional stimuli (amino acids and insulin) along with physical activity are the major acute physiological regulators of muscle protein turnover and are responsible for its diurnal oscillations and overall muscle protein net balance [39]. In the present study, we examined how aging in men and women affects the rate of muscle protein synthesis during basal, postabsorptive conditions and during a hyperaminoacidemic-hyperinsulinemic-euglycemic clamp. We discovered that both old men and old women exhibit anabolic resistance to nutritional stimuli; in addition, the basal rate of muscle protein synthesis is increased in old women compared with young women and young and old men. Aging therefore affects muscle protein synthesis differently in men and women, and men and women need to be considered separately when evaluating the effect of aging on muscle protein synthesis.

Only a limited number of studies have been conducted to investigate potential sexual dimorphism in muscle protein turnover to date. Most of them were limited to young men and women only and found, as we did in the present study, no differences in muscle protein turnover between them [7-11]. In another study we conducted, we found major differences in muscle protein synthesis rates between old men and women [12]; however, the fact that subjects in this study were obese has been criticized as a major confounding variable. Only one study so far evaluated the effect of both sex and age on muscle protein turnover [13] and found no age by sex interaction in the basal rate of muscle protein synthesis. However, this study [13] was limited to basal, postabsorptive conditions only and included only old men with hypogonadism and old women with low serum dehydroepiandrosterone concentration, which may have confounded the results. Hypoandrogenemia is associated with a reduced lean body mass [14] and treatment with testosterone increases the muscle protein synthesis rate [15-20]. In the present study, plasma androgen concentrations were within the normal range for all subjects.

Consistent with the results from many earlier studies which focused on the effect of aging but included only men or both men and women without analyzing them separately [30,40-47], we found no difference in the basal, postabsorptive muscle protein FSR in healthy young and old men and a blunted anabolic response to nutritional stimuli in old compared with young subjects (both men and women). The basal rate of muscle protein synthesis, however, was greater in our old compared with our young women and young and old men. This is consistent with what we have previously observed in obese older adults [12] but contradicts the only other study we are aware of that specifically evaluated the effect of aging on muscle protein synthesis in women [48]. Chevalier et al. [48] report no difference between young and old women in the basal rate of muscle protein synthesis and the muscle protein synthesis rate during a simulated fed state. The reason(s) for the discrepancy are not entirely clear but could be related to the amount of amino acids provided. Chevalier et al. [48] raised the plasma leucine concentration during the clamp to ~250% above basal values, which is equivalent to the peak increase after a maximally stimulating dose of protein (30 g) [49] or amino acids [40]. The clamp in our study, on the other hand, was designed to achieve plasma insulin and amino acid concentrations equivalent to those seen after ingestion of ~22 g of casein or soy protein or consumption of a 2300–3300 kJ mixed nutrient meal containing ~26 g protein and ~70 to 90 g carbohydrates [50-52]; plasma leucine concentration during the clamp in our study was therefore increased to only ~ 40% above basal values. If this is indeed the main reason for the difference in results between our study and the one by Chevalier et al. [48], it would suggest that old women may require a large amount of protein to respond adequately to nutritional stimuli. Old men, on the other hand, seem to be unable to benefit from the consumption of more protein/amino acids [40]. It has also been proposed that differences in the availability of leucine per se, which is thought to be a major regulator of muscle protein synthesis [32], might be a key factor responsible for whether or not there is anabolic resistance in older adults [53]. However, plasma leucine concentration in our study was not different between young and old men and not different between young and old women and can therefore not help explain the anabolic resistance of muscle in older adults. Moreover, plasma leucine concentration was ~20% lower in women (both young and old) than in men, which is consistent with earlier work from our own group [12] and by others [54] but again does not help explain the differences in muscle protein synthesis rates between groups in our study. The reason(s) for the difference in plasma leucine concentration is currently unclear.

The mechanism(s) responsible for the faster basal muscle protein FSR in our old women and why aging affects the basal muscle protein synthesis rate differently in men and women is unclear. One potential explanation may be that the menopause-induced decline in estradiol and progesterone concentrations leads to an increase in the basal rate of muscle protein synthesis. In rodents, surgically-induced menopause (ovariectomy) increases the rate of muscle protein synthesis and replacement of either estrogen or progesterone prevents this effect [55]. Whether the age-related declines in estrogens and/or progesterone similarly affect muscle protein turnover in women is not known; accordingly, it is also not known whether estrogens and/or progesterone are potentially important regulators of muscle protein metabolism in men.

In line with the muscle protein synthesis data in the present study, we have recently reported no difference in anabolic signaling between young men and young women [11] and several other investigators have reported a blunted nutrient-induced increase in mTOR signaling in old compared with young subjects [40,42]. In the present study, we therefore chose to focus on factors that could potentially help explain the greater basal rate of muscle protein synthesis in old women compared with young women and young and old men and possibly provide some insight into the anabolic resistance of older adults. To this end, we evaluated the muscle mRNA expression of the myogenic regulatory factor myoD, and the plasma concentration and muscle mRNA expression of the muscle growth inhibitor myostatin and follistatin. Myostatin is a muscle growth inhibitor which is produced primarily in skeletal muscle cells, circulates in the blood and acts on muscle tissue by blocking genes induced during differentiation (e.g., myoD [21]) and by inhibiting the anabolic signaling cascade and muscle protein synthesis [22-26]. Follistatin is ubiquitously expressed, circulates in the blood and binds to and thereby inhibits myostatin [27,28]. We found that plasma follistatin concentration was, paradoxically, greater in old than young subjects but not different in men and women whereas plasma myostatin concentration and muscle myoD, myostatin and follistatin mRNA expressions were not different in men and women and not affected by aging. These findings are generally consistent with those reported by others [56-59] and do not match the differences in muscle protein turnover between young and old men and women. We recognize that our muscle mRNA expression data may provide only limited information; however, in pilot experiments, we were not able to identify antibodies specific for myoD and myostatin that passed rigorous quality control criteria. The exact mechanism(s) of myostatin action (e.g., via plasma or locally within muscle or both) are not entirely clear and few studies have compared muscle and plasma myostatin concentrations. Nevertheless, those that did, show good qualitative agreement between the plasma myostatin concentration and myostatin protein expression in human muscle [60,61].

The higher basal muscle protein FSR in old women is not inconsistent with a reduced muscle mass in old compared with young women because muscle mass is determined by the net balance between muscle protein synthesis and muscle protein breakdown. In fact, very high muscle protein synthesis rates are often observed in extremely catabolic conditions such as major burns because both muscle protein synthesis and muscle protein breakdown rates are upregulated but the increase in muscle protein breakdown exceeds the increase in muscle protein synthesis resulting in net muscle protein loss despite an increase in muscle protein synthesis [62]. Our data therefore suggest that accelerated muscle protein breakdown may be a major contributor to the age-associated loss of muscle mass in older women.

We measured the global/mixed muscle protein synthesis rate and it is therefore possible, but unlikely, that our results are not applicable to myofibrillar proteins, which account for the bulk of muscle proteins. During basal, postabsorptive conditions at rest, there is very good correlation between the mixed and the myofibrillar protein FSR in both young and older subjects [63-65]. Furthermore, the increases in myofibrillar, sarcoplasmic and mitochondrial protein synthesis rates in response to hyperaminoacidemia/hyperinsulinemia mirror each other [40,66-68].

In summary, we report that healthy aging is associated with an increase in the basal rate of muscle protein synthesis in women and resistance to the anabolic effect of nutritional stimuli in both men and women. These findings indicate that there is sexual dimorphism in the age-related changes in muscle protein synthesis and the metabolic processes responsible for the age-related decline in muscle mass.

## Conclusion

There are no differences in the rates of muscle protein synthesis in young men and young women but there is sexual dimorphism in the age-related changes in muscle protein synthesis. Men and women therefore need to be considered separately when evaluating muscle protein synthesis rates in older adults.

## Abbreviations

ANOVA: analysis of variance; BSA: body surface area; FFM: fat-free mass; FSR: fractional synthesis rate; GC-MS: gas chromatography mass spectrometry; HOMA-IR: homeostasis model assessment of insulin resistance; Ra: rate of appearance; Rd: rate of disappearance; RNA: ribonucleic acid; *t*-BDMS: *t*-butyldimethylsilyl; TTR: tracer-to-tracee ratio.

## Competing interests

The authors declare that they have no competing interests.

## Authors’ contributions

GIS carried out the experiments, processed muscle samples, analyzed the data and drafted the manuscript. DNR was responsible for the medical aspects of the study and obtained the biopsy samples. AMH, KTC, and BNF were involved in processing the study samples and interpretation of the data. BM designed the study and obtained funding for the study, supervised the experiments, sample processing and data analyses and prepared the final version of the manuscript. All authors read and approved the final manuscript.

## Sources of funding

This publication was supported by NIH grants AR 49869, HD57796, DK87821, UL1 RR024992 (Washington University Clinical Translational Science Award), RR 00954 (Biomedical Mass Spectrometry Resource), and DK 56341 (Nutrition and Obesity Research Center), and a grant from the Longer Life Foundation. Kari Chambers was supported by an American Liver Foundation Liver Scholar Award and Dominic Reeds was supported by an American Society of Nutrition Physician Nutrition Support Specialist Award.
